# Genome sequences of *Colletotrichum eleusines* and *C. echinochloae*: two pathogens from *Poaceae* weeds

**DOI:** 10.1128/MRA.00722-23

**Published:** 2024-02-08

**Authors:** Qiongnan Gu, Shihai Chu, Qichao Huang, Lin Li, Anan Chen, Tom Hsiang, Ruhai Li

**Affiliations:** 1 Institute of Plant Protection and Soil Fertilizer, Hubei Academy of Agricultural Sciences/Key Laboratory of Integrated Pest Management on Crops in Central China, Ministry of Agriculture/Hubei Key Laboratory of Crop Diseases, Insect Pests and Weeds control, Wuhan, Hubei, China; 2 School of Environmental Sciences, University of Guelph, Guelph, Canada; University of California Riverside, USA

**Keywords:** *Colletotrichum*, anthracnose, comparative genomics, nanopore sequencing, Illumina, glomerella

## Abstract

We present two whole-genome sequences of *Colletotrichum* strains which were isolated from *Eleusine indica* and *Echinochloa crus-galli* using Nanopore and Illumina technologies, as part of screening for potential mycoherbicide. The genome sequences will provide important genetic information and will be useful for further research into secondary metabolites of *Colletotrichum*.

## ANNOUNCEMENT

*Colletotrichum eleusines* isolate was obtained from diseased goosegrass plants of Hubei province, China (30°17′24″N, 114°07′56″ E, 30 m above sea level). *C. echinochloae* isolate was obtained from diseased barnyardgrass plants of Hubei province, China (32°7′49″N, 112°20′35″ E, 91 m above sea level). All strains were isolated through single spore isolation, and their pathogenicity on the host was confirmed by the Koch’s postulates (1, 2). The strains were incubated at 25°C on PDA medium for 7 days and verified by observation of morphology, concatenated ITS, Sod2, Apn2, and Mat1-2 sequences (2).

All isolates used in this study were cultivated in potato dextrose broth for 7 days, and genomic DNA was extracted from fresh mycelial tissue using the CTAB method (3) further purified with the Wizard Genomic DNA purification Kit (Promega, Madison, WI, USA). The high-molecular weight genomic DNA (gDNA) was obtained by removing fragments shorter than 2 kbp using the Agencourt AMPure XP (Beckman Coulter Inc., USA). The gDNA was repaired using the PreCR Repair Mix (New England Biolabs, Ipswich, MA, USA) before subjected to library construction. Both *Colletotrichum* genomes were sequenced and assembled using a combination of long and short reads. Long reads were generated first by using an Oxford Nanopore Technologies Ligation Sequencing Kit (SQK-LSK109, Oxford Nanopore Technologies, Oxford, UK), and then, a library of each strain was sequenced with primed R9.4 Spot-On Flow cells for 60 h on a PromethION sequencer (Oxford Nanopore Technologies). Short reads were generated using genomic DNA libraries constructed with an NEBNext UltraTM Prep Kit (150-bp paired end reads, with a read length of 500 bp) and sequenced on the Illumina NovaSeq 6000 platform (Illumina Inc., San Diego, California, USA). The Oxford Nanopore Technologies (ONT) reads fastq data were basecalled using Guppy v.3.03 (HAC model). FastQC was used for quality control for Illumina reads. ONT reads with a *Q*-score lower than 7 were filtered out. The long-read Nanopore sequences were assembled with NECAT v.20200119 (4), and raw reads of ONT were mapped to the draft assembly using minimap2 v.2.11 (5), and two rounds of polishing were performed with Racon v.1.4.11 (6). To further improve assembly, Illumina short reads were mapped to the assembly using BWA v.0.7.17 (7) and two rounds of polishing were performed with Pilon v.1.23 (8). Default parameters were used except where otherwise noted.

The nuclear genome of *C. eleusines* CCTCC2020688 consisted of 15 contigs with a total assembled length of 53.51 Mbp, 51.11% GC content, and a maximum contig size of 6,664,387 bp (Table 1). In comparison, the nuclear genome assembly of *C. echinochloae* CCTCC2020689 consisted of 21 contigs with a total assembled length of 62.25 Mbp, 46.59% GC content, and a maximum contig size of 8,328,496 bp. Assembly completeness was assessed with the program BUSCO (v.3.0.1) (9), which estimated the gene complement to be 99.3% complete for both *C. eleusines* and *C. echinochloae* genomes, using a 290-gene database (fungi_odb9).

**TABLE 1:** Summary statistics of *C. eleusines* and *C. echinochloae* genomes

Statistics		
Variables	*C. eleusines*	*C. echinochloae*
Isolate	CCTCC2020688	CCTCC2020689
NCBI accession	JADEZO000000000	JADEZT000000000
Sequencing technology	ONT PromethION & Illumina NovaSeq 6000	ONT PromethION & Illumina NovaSeq 6000
Genome assembly		
Assembly size (bp)	53,507,824	62,246,358
Number of contigs	15	21
Contig N50 length (bp)	5,135,075	5,346,453
Contig N90 length (bp)	2,723,754	3,463,513
GC (%)	51.11	46.59
Average contig length (bp)	3,567,188.27	2,964,112.29
Longest contig (bp)	6,664,387	8,328,496
Shortest contig (bp)	33,194	25,553
Total reads for ONT (bp)	48,350,746	610,642
Read length N50 for Nanopore (bp)	25,147	20,000
Total reads for Illumina (bp)	48,350,746	55,323,334
Average depth for Illumina	133.77	132.23
Genome completeness		
Complete BUSCOs	288	288
Complete and single BUSCOs	288	288
Complete and duplicated BUSCOs	0	0
Fragmented BUSCOs	1	1
Missing BUSCOs	1	1

## Data Availability

The genome sequences described in this article have been deposited in DDBJ/ENA/GenBank under the accession number JADEZO000000000 for *C. eleusines* CCTCC2020688 (BioProject: PRJNA671855; Biosample: SAMN16552423), [the version described in this paper is the first version (JADEZO000000000.1), and the raw sequencing reads are available under SRA accession numbers SRX9363665 and SRX9363664, respectively] and JADEZT000000000 for *C. echinochloae* CCTCC2020689 (BioProject: PRJNA672254; Biosample: SAMN16551125) [the version described in this paper is the first version (JADEZT000000000.1), with raw sequencing reads available under SRA accession numbers SRX9369587 and SRX9369586, respectively].
